# Seasonal Effect of a Small Hydropower Plant on Benthic Biofilm Microbial Community Structure and Predicted Functional Potential

**DOI:** 10.1002/ece3.74330

**Published:** 2026-09-08

**Authors:** Huimin Gao, Sangar Khan, Xinxin Qi, Zongwei Lin, Guohao Liu, Yuanyuan Lv, Min Zhang, Zhijun Xia, Naicheng Wu

**Affiliations:** ^1^ School of Geography and Remote Sensing, Zhejiang‐Germany Joint Laboratory on Remote Sensing of Coastal Ecosystem Ningbo University Ningbo China; ^2^ Department of Geosciences and Geography University of Helsinki Helsinki Finland; ^3^ Leibniz Institute of Freshwater Ecology and Inland Fisheries Berlin Germany; ^4^ State Key Laboratory of Water Cycle and Water Security China Institute of Water Resources and Hydropower Research Beijing China; ^5^ School of Civil Engineering and Future Cities, Institute of Hydraulic and Ocean Engineering Ningbo University Ningbo China; ^6^ Department of Hydrology and Water Resources Management Kiel University Kiel Germany

**Keywords:** assembly mechanisms, benthic biofilm microbial communities, niche characteristics, periphyton, predicted functional potential, small hydropower plants

## Abstract

Benthic biofilm microbial communities play a critical role in riverine primary production, nutrient cycling, and organic matter decomposition, yet their responses to hydrological alterations due to small hydropower plants (SHPs) remain poorly understood, particularly with respect to seasonal dynamics. This study conducted a one‐year monitoring survey across four river sections (upstream, reservoir, dewatered reach, and downstream) in the Oujiang River Basin, Zhejiang Province, China, covering all four seasons. The results showed that the reservoir section exhibited distinct bacterial composition, while the dewatered reach harbored the highest diversity, broader niche breadth, and was governed by stochastic assembly processes. Seasonally, deterministic processes dominated in summer, whereas stochastic processes prevailed in winter. Predicted functional profiling suggested that the SHP may exert a regulatory role in carbon and nutrient metabolism, with pronounced functional differences observed in summer. This study indicates that SHPs reshape microbial assembly and predicted function by altering hydrological regimes. As an observational case study based on a single representative facility, these findings should be interpreted as hypothesis‐generating rather than conclusive; however, they provide key ecological insights and a methodological reference for future impact assessments of similar diversion‐type SHPs in subtropical river systems.

## Graphical abstract

Biofilm microbial composition varies significantly; diversity is higher in spring and autumn. The dewatered reach shows the broadest niche and dominant stochastic assembly. Seasonal assembly shift: deterministic in summer, stochastic in winter. Nutrient cycling genes vary more among sites than seasons under SHP influence.

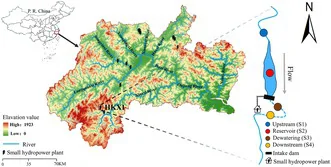

## 1 Introduction

Hydropower is an advanced technology built upon over a century of development for clean energy production (Ellabban et al. 2014; Lees et al. 2016). Currently, hydropower accounts for approximately 20% of global electricity generation, contributing significantly to meeting both baseload and peak‐load demand (Akpinar et al. 2011; Kahn et al. 2014). However, the construction of hydropower facilities has led to the fragmentation of two‐thirds of the world's major rivers (Nilsson et al. 2005). For instance, the Yangtze River has over 50, 000 dams, which not only generate electricity but also support irrigation (Wu et al. 2019). This fragmentation has direct implications for hydrological characteristics, river system connectivity, fluvial geomorphology, and the ecological requirements of microbial communities, and also indirectly affects regional biodiversity by exacerbating habitat loss and degradation (Lees et al. 2016). Research indicates that freshwater biodiversity is declining sharply due to habitat fragmentation and degradation (Dudgeon et al. 2006).

The effect of hydropower stations on regional biodiversity depends on their size and type (He et al. 2024). In China, small hydropower plants (SHPs) are defined as those with an installed capacity below 50 MW, and they play a dominant role in the global SHP sector (Yu et al. 2021). SHPs have been promoted for their ability to alleviate electricity shortages, manage water resources, and support regional development (Lange et al. 2018; Yu et al. 2021). They are also widely perceived to have lesser ecological impacts than large hydropower projects (Couto and Olden 2018). Recent studies indicate that the ecological consequences of SHPs may be underestimated (Couto and Olden 2018; Hedger et al. 2025). Current studies on aquatic biodiversity under the influence of SHPs have predominantly focused on macro‐organisms such as fish, macroinvertebrates, and algae (Larinier 2008; Lin et al. 2024; Qi et al. 2024), with comparatively less attention being given to microbial communities within benthic biofilms (Gao et al. 2025).

Approximately 40%–80% of all microbial cells on Earth exist within biofilms, making them the predominant form of microbial life in aquatic environments (Costerton et al. 1995; Flemming and Wuertz 2019). In freshwaters, hydrological dynamics profoundly influence biofilm structure and function by regulating shear stress and material transport (Quan et al. 2022). The intricate network of channels and pores within biofilms not only facilitates the transport and storage of nutrients but also serves to buffer against environmental stresses (Schramm and de Beer 1999). Their productivity and diversity are strongly influenced by the nutritional status of the water body (Lourenço‐Amorim et al. 2014). Therefore, investigating the response and underlying mechanisms of benthic biofilm microbial communities to the influence of SHPs is of considerable importance.

The construction of SHPs diminishes the longitudinal connectivity of rivers, resulting in highly fragmented and heterogeneous habitats over short distances (Couto and Olden 2018; He et al. 2024). Previous studies have revealed that the microbial community structure and diversity of the benthic biofilm in the reservoir section exhibit greater variation compared to other sampling sites (Gao et al. 2025). The dewatered reach, maintained by ecological flow, is a result of intense SHP disturbance and serves as a critical zone connecting the reservoir and downstream sections for microbial community assembly (Yu et al. 2021). It is likely to exhibit community assembly processes distinct from those in the upstream section, which remains unaffected by the SHP. The impacts of climate change primarily manifest through alterations in hydrological conditions (Maran et al. 2014), and thus, the habitat fragmentation and seasonal extremes created by SHPs provide a natural experiment to dissect how niche and neutral processes jointly sculpt microbial community assembly across the disturbed river corridor. Both niche‐based and neutral theories have been proposed as fundamental frameworks explaining the assembly, dynamics, and structure of ecological communities (Tilman 2004). A growing body of research in microbial ecology supports the integrated application of these theories to unravel microbial community assembly, thereby providing mechanistic insights into diversity patterns and species composition (Bissett et al. 2010; Burke et al. 2011; Stegen et al. 2013; Zhao et al. 2024). The co‐occurrence of niche and neutral processes during assembly indicates that bacterial communities with distinct traits may be governed by different mechanisms (Langenheder and Székely 2011; Pandit et al. 2009). Differences in community structure and assembly processes may further manifest in functional adaptations of the microbial communities, such as functional potential, due to its close link to environmental adaptation (Kashtan et al. 2014; Shi and Falkowski 2008). The present study therefore builds upon these previous findings (Gao et al. 2025; Lin et al. 2024) by incorporating seasonal monitoring, ecological niche and neutral model frameworks, and functional gene prediction to provide a more mechanistic understanding of SHP impacts on benthic biofilm microbial communities.

Against this backdrop, we aim to address the following research questions: (I) How do the composition and diversity of benthic biofilm microbial communities vary across seasons and sites under SHP influence? (II) What are the impacts of SHPs on the niche characteristics of these communities in different seasons and at different sites? (III) How do the assembly processes of benthic biofilm microbial communities differ spatially and temporally? (IV) Do SHPs induce significant changes in the predicted functional potential profiles of benthic biofilms across different sites? Building on current knowledge, we hypothesize that: (1) Under the SHP influence, benthic biofilm microbial communities differ significantly among sites in terms of community structure, diversity, assembly processes, and predicted functional potential, with niche and neutral processes differing most markedly between upstream and dewatered reaches, and with the reservoir section showing the greatest deviation from others. (2) On a temporal scale, these communities exhibit pronounced seasonal variation in all the above ecological attributes, particularly between the wet (summer) and dry (winter) seasons.

## 2 Materials and Methods

### 2.1 Study Area

**FIGURE 1:**
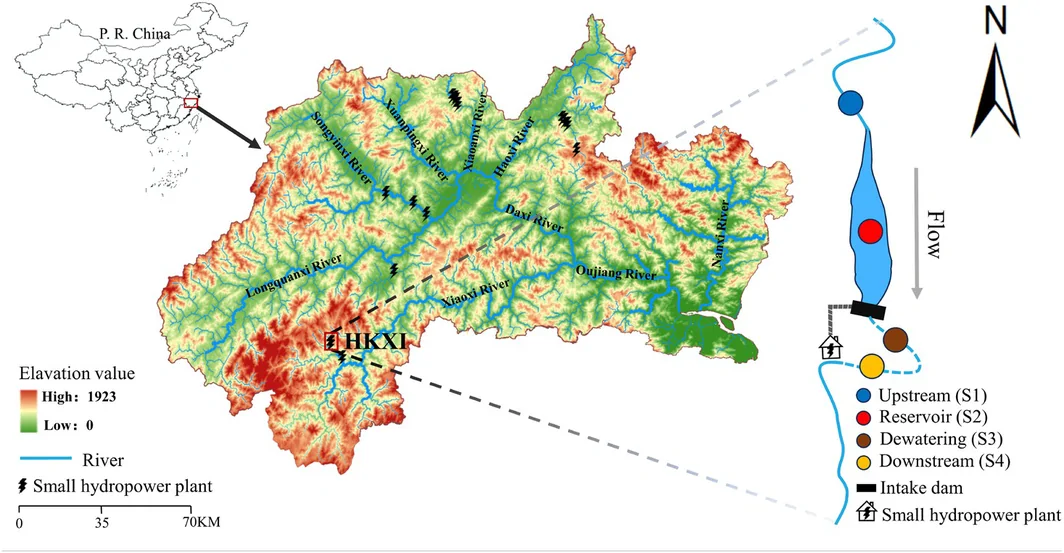
The Distribution of the Huangkengxu No. 1 Small Hydropower Plant in the Lishui section of the Oujiang River Basin, China.

The present study was conducted at the Huangkengxu No. 1 SHP in the upper reaches of the Oujiang River Basin, Zhejiang Province, China. The study region is located in Lishui City, a core hydropower‐rich area that accounts for approximately 40% of Zhejiang's developable hydropower capacity and hosts nearly 800 operational small hydropower facilities, representing a typical region of intensive SHP development in subtropical China (Qi et al. 2024). The Oujiang River features a typical subtropical monsoon climate with distinct dry–wet seasonal alternations and prominent hydrological fluctuations, which are widespread characteristics of small river basins affected by low‐head diversion‐type hydropower stations (Wei et al. 2012), with an average gradient of 3.4°, an elevation of 1300 m, and abundant hydropower resources, boasting a potential power generation capacity of 22.6 billion kWh (Lin et al. 2024). The region experiences a subtropical monsoon climate, characterized by strong seasonal precipitation that results in distinct dry (winter) and wet (summer) seasons. The Huangkengxu No. 1 SHP (119°23′08″–119°23′19″ E and 27°54′47″–27°55′23″ N), is located in the upper reaches of the Oujiang River Basin (Figure 1). It has a minimum ecological flow requirement of 0.0536 m^3^s^−1^, reduces the flow in a 1.3 km dewatered river reach, and has an installed capacity of 800 kW (2 × 400 kW units). The catchment area above the dam is 26.3 km^2^, and the plant generates 3.08 million kWh annually. This SHP forms a typical segmented river hydrological pattern, which reflects the representative hydrological and ecological alterations induced by small hydropower operations. Additionally, the study watershed is dominated by forested land cover with limited anthropogenic interference, which helps isolate SHP‐induced ecological effects from confounding human disturbances. Given its typical hydrological regime, standard diversion‐type structure, and climatic background consistent with most regional SHPs, this station can serve as a representative case for exploring the ecological impacts of small hydropower development. Accordingly, findings from this case study provide targeted and referential implications for understanding microbial ecological responses to SHP disturbance in similar subtropical river systems, rather than making broad generalizations to all SHPs worldwide.

### 2.2 Field Sampling and Processing

The area was monthly sampled from July 2023 to May 2024. Sampling in June 2024 was not possible due to the high flooding conditions; data from 11 months were used for the final analysis. To analyze the temporal impact of the SHP on benthic biofilm microbial communities, the 11 months were categorized into four distinct seasons. This seasonal division aligns with the local hydrological regime, with summer being the primary wet season and winter the primary dry season. Four sampling sites were established for monthly collection of benthic biofilm microbial samples: Upstream reach (S1; a natural flowing reach unaffected by the SHP), Reservoir (S2; located within the impoundment area of the SHP), Dewatered reach (S3; situated below the dam, characterized by controlled ecological flow that maintains limited fluvial conditions), and Downstream reach (S4; located in the flowing water environment immediately upstream of the power plant outlet) (Cai 2026). Benthic biofilm samples were collected from these four sites—upstream, reservoir, dewatered reach, and downstream—of the Huangkengxu No. 1 SHP. It should be noted that the four sampling sites (S1–S4) were distributed along a longitudinal gradient within the same river channel, where natural downstream variation in physicochemical parameters and community composition may inherently exist. Therefore, while we attribute the differences among these sites primarily to SHP operation, we acknowledge that a portion of the observed variation could also reflect baseline longitudinal gradients unrelated to dam effects. Due to the small size of the facility, all sites were accessible by walking, including the reservoir site (S2), which featured sufficiently shallow water levels during water release periods for safe access. At each site, three to five rocks representing distinct microhabitats were selected. Biofilms (*n* = 3) were scrubbed from the rock surfaces using sterile brushes and distilled water, and then transported to the laboratory for filtration through glass fiber filters (0.45 μm pore size). All samples were stored at −80°C until shipment to Shanghai OE Biotech Co. Ltd. for subsequent molecular processing. At each sampling site and month, a suite of hydrological and physicochemical parameters was monitored. River width and depth were determined with a rangefinder. Flow velocity was measured with a Global Water flow meter. Key water quality variables, including water temperature (WT), dissolved oxygen (DO), total dissolved solids (TDS) and pH, were recorded in situ using a YSI handheld meter (Model YSI‐6600 V2, USA). Furthermore, laboratory analysis was conducted for total nitrogen (TN), total phosphorus (TP), nitrate nitrogen (NO_3_‐N), and ammonia nitrogen (NH_3_‐N) in accordance with the Methods for Monitoring and Analysis of Water and Wastewater (State Environmental Protection Administration 2002). Specifically, TN was determined by alkaline potassium persulfate digestion–UV spectrophotometry; TP was determined by ammonium molybdate spectrophotometry; NO_3_‐N was determined by phenol disulfonic acid spectrophotometry; and NH_3_‐N was determined by distillation–neutralization titration.

### 2.3 16S rRNA Gene Sequencing

Genomic DNA of prokaryotes was extracted from samples collected at different sites using the MagPure Soil DNA LQ Kit (Magan Company). The concentration and purity of the extracted DNA were measured with a NanoDrop 2000 spectrophotometer (Thermo Fisher Scientific, USA), and its purity was assessed by agarose gel electrophoresis. The V3–V4 hypervariable region of the 16S rRNA gene was amplified using barcoded primers 343F (5′‐TACGGRAGGCAGCAG‐3′) and 798R (5′‐AGGGTATCTAATCCT‐3′) with Takara ExTaq high‐fidelity DNA polymerase in a polymerase chain reaction (PCR) (Nossa et al. 2010). The amplification products were verified by agarose gel electrophoresis, purified using AMPure XP magnetic beads, and then subjected to a second round of amplification and repurification. The purified amplicons were accurately quantified with a Qubit fluorometer, normalized to a standard concentration, and prepared for subsequent sequencing.

Sequencing was performed on the Illumina NovaSeq 6000 platform to generate 250‐bp paired‐end reads. Raw sequencing data in FASTQ format were processed as follows: primer sequences were removed using Cutadapt, and quality control, denoising, read merging, and chimera removal were performed with the DADA2 pipeline (default parameters) within the QIIME2 framework. This process yielded representative sequences and an amplicon sequence variant (ASV) abundance table. Taxonomic assignment was performed by aligning the representative sequences against the SILVA database (version 138) using the q2‐feature‐classifier plugin in QIIME 2 (Bolyen et al. 2019; Callahan et al. 2016). All library preparation and sequencing procedures were conducted by Shanghai OE Biotech Co. Ltd.

### 2.4 Data Analysis

Before statistical analysis, monthly ASV abundances were averaged within each season per site, yielding 16 unique season–site combinations for subsequent analyses. Therefore, “Season” and “Site” were treated as fixed factors, while “Month” was not included as an independent factor in the statistical models. Alpha diversity, beta diversity (based on Bray‐Curtis distances), and permutational multivariate analysis of variance (PERMANOVA) were performed in R (version 4.5.0) with the “vegan” package, and non‐metric multidimensional scaling (NMDS) was used to visualize community structure (Eskelinen and Harrison 2015; Mousing et al. 2016; Oksanen et al. 2015). Levins' niche breadth index and niche overlap coefficient were computed using the “spaa” package in R (Zhang et al. 2016). To distinguish generalists and specialists within the bacterial communities, a permutation algorithm was applied at the amplicon sequence variant (ASV) taxonomic level using the “EcolUtils” package in R (Levins 1968; Losos et al. 2003; Wang et al. 2024). This algorithm randomizes species occurrence frequencies to generate null distributions. ASVs with niche breadth indices exceeding the 95% upper confidence limit of the null distribution were classified as generalists, while those below the 95% lower confidence limit were classified as specialists. The remaining ASVs within the confidence interval were defined as neutral taxa. A neutral community model (NCM) was fitted to evaluate the relative contributions of deterministic and stochastic processes in bacterial community assembly. The results were further validated by calculating the modified stochasticity ratio (MST) using the “NST” package in R (Dini‐Andreote et al. 2015; Ning et al. 2019).

To investigate the expression of functional genes in benthic biofilm microbial communities across different sites, PICRUSt2 (version 2.3.0b0) was employed to predict the abundance of KEGG orthologs (KOs) from the sequencing data. The “pathview” package in R was used to visualize the predicted functional profiles by mapping them onto KEGG pathway maps, thereby identifying differences in predicted functional potential related to carbon fixation, nitrogen, and sulfur metabolism across sites (Luo and Brouwer 2013). To identify enzymes and genes involved in metabolic pathways that differed significantly among sites and seasons, Welch's *t*‐test was performed for pairwise comparisons across multiple sample groups, and the resulting *p*‐values were adjusted using the Benjamini–Hochberg (BH) method to control the false discovery rate (FDR); adjusted *q*‐values < 0.05 were considered statistically significant. Gene abundance was subsequently annotated on pathway diagrams according to distinct groups and color‐coded accordingly (Zheng et al. 2025).

Relationships between environmental conditions and microbial diversity were examined using Pearson correlation analysis and structural equation modeling (SEM). Some variables were not recorded at S2 and S3 in August and October (four of 44 records, 0.7% of the environmental matrix). These values were imputed by linear interpolation between adjacent monthly measurements at the same site; no records were excluded. Table S1 has been updated to show the imputed values, which are flagged as such. A sensitivity analysis using complete cases only (*n* = 40) returned equivalent results, with all path coefficients retaining their sign and significance (Table S4).

Unlike the community composition and assembly analyses described above, which used seasonally averaged data, both analyses were conducted on the full set of monthly records (*n* = 44 site‐month observations; four sites × 11 months), to retain the temporal resolution required to characterize environmental variability. Pairwise Pearson correlations among all hydrological, physico‐chemical, nutrient and diversity variables were calculated using the “psych” package and visualized with “corrplot”; correlation coefficients (r) and associated *p* values are reported in Table S2.

SEM was used to test the hypothesized causal structure in which hydrological conditions influence microbial diversity both directly and indirectly through physico‐chemical conditions and nutrient concentrations. Because the number of site‐month observations was modest relative to the number of measured variables, each conceptual block was represented by a composite variable rather than a latent variable with freely estimated loadings, thereby limiting the number of estimated parameters. Composites were derived as the first principal component of their constituent indicators, standardized to unit variance: hydrology (river width, depth), physico‐chemical conditions (pH, WT, DO, TDS), nutrients (TN, TP, NO_3_‐N, NH_3_‐N), and microbial diversity (Shannon, Chao1, Simpson). The resulting structural model comprised five directed paths and nine estimated parameters, giving a ratio of observations to parameters of approximately 4.9:1.

Models were fitted by maximum likelihood using the “lavaan” package in R (version 4.5.0). Model adequacy was evaluated using the model chi‐square statistic, the comparative fit index (CFI), the Tucker–Lewis index (TLI), the standardized root mean square residual (SRMR), and the root mean square error of approximation (RMSEA), with a nonsignificant chi‐square (*p* > 0.05), CFI > 0.95 and, SRMR < 0.08 taken to indicate acceptable fit. RMSEA is reported for completeness but was not used as a primary criterion, as it is positively biased in models with low degrees of freedom and small samples (Kenny et al. 2015). Standardized path coefficients are reported with their standard errors and exact *p* values (Table S3b). Before interpretation, the assumptions underlying each structural equation were assessed: linearity from residual‐versus‐fitted plots, multicollinearity from variance inflation factors, and residual normality using the Shapiro–Wilk test (Table S3c).

River depth was not recorded at S2 and S3 in August and October (four of 44 site‐month records). A sensitivity analysis restricted to complete cases (*n* = 40) returned equivalent results, with all path coefficients retaining their sign and significance classification (Table S4).

## 3 Results

### 3.1 Composition and Diversity of Benthic Biofilm Microbial Communities

Hydrological and physico‐chemical conditions across the four sites and four seasons are summarized in Table 1. Hydrological conditions differed markedly among sites, with the reservoir section (S2) being the deepest (62.44 ± 16.55 cm) and the dewatered reach (S3) the widest (17.79 ± 1.80 m; width F = 206.57, *p* < 0.001; depth F = 12.27, *p* < 0.001). In contrast, most water chemistry variables showed no significant spatial differences: among sites, only pH (F = 5.46, *p* = 0.003) and NO_3_‐N (F = 4.04, *p* = 0.013) varied significantly, whereas WT, DO, TDS, TN, TP, and NH_3_‐N were not significant (all *p* > 0.05).

**TABLE 1:** Hydrological and physico‐chemical characteristics of the four sampling sites across the four seasons (mean ± SD, *n* = 44 site‐month observations).

(a) Among sites						
Variable	S1	S2	S3	S4	F	*p*
River width (m)	5.08 ± 1.78	13.74 ± 1.60	17.79 ± 1.80	2.92 ± 1.28	206.57	< 0.001
Water depth (cm)	35.09 ± 12.36	62.44 ± 16.55	35.22 ± 11.53	28.64 ± 12.22	12.27	< 0.001
Water temperature (°C)	17.39 ± 5.29	17.10 ± 5.30	16.90 ± 5.29	17.56 ± 5.86	0.03	0.992
Dissolved oxygen (mg L^−1^)	10.48 ± 2.41	9.82 ± 2.66	9.69 ± 2.28	10.36 ± 2.43	0.28	0.839
pH	6.73 ± 0.48	7.28 ± 0.41	7.27 ± 0.32	7.31 ± 0.34	5.46	0.003
Total dissolved solids (mg L^−1^)	12.59 ± 1.89	12.82 ± 1.70	12.94 ± 1.85	13.35 ± 1.65	0.36	0.782
Total nitrogen (mg L^−1^)	0.75 ± 0.41	0.66 ± 0.31	0.75 ± 0.28	0.65 ± 0.29	0.31	0.821
Total phosphorus (mg L^−1^)	0.061 ± 0.016	0.072 ± 0.018	0.068 ± 0.026	0.050 ± 0.017	2.58	0.067
NO_3_‐N (mg L^−1^)	0.38 ± 0.15	0.26 ± 0.10	0.25 ± 0.06	0.25 ± 0.10	4.04	0.013
NH_3_‐N (mg L^−1^)	0.25 ± 0.20	0.20 ± 0.13	0.19 ± 0.11	0.20 ± 0.12	0.38	0.767

(b) Among seasons						
Variable	Spring	Summer	Autumn	Winter	F	*p*
River width (m)	9.73 ± 6.56	11.68 ± 6.31	9.95 ± 6.69	8.76 ± 6.46	0.32	0.809
Water depth (cm)	40.42 ± 14.01	40.17 ± 14.57	36.20 ± 22.39	41.00 ± 20.91	0.14	0.934
Water temperature (°C)	17.57 ± 3.48	23.99 ± 1.47	19.01 ± 1.73	10.64 ± 2.69	47.21	< 0.001
Dissolved oxygen (mg L^−1^)	9.47 ± 1.05	7.49 ± 0.68	9.08 ± 0.90	13.44 ± 1.20	68.06	< 0.001
pH	7.15 ± 0.47	7.17 ± 0.22	7.42 ± 0.35	6.86 ± 0.50	3.76	0.018
Total dissolved solids (mg L^−1^)	11.81 ± 1.10	13.49 ± 1.50	13.54 ± 1.56	13.05 ± 2.16	2.77	0.054
Total nitrogen (mg L^−1^)	0.97 ± 0.14	0.61 ± 0.27	0.43 ± 0.16	0.77 ± 0.37	10.20	< 0.001
Total phosphorus (mg L^−1^)	0.062 ± 0.018	0.082 ± 0.018	0.066 ± 0.022	0.047 ± 0.013	6.43	0.001
NO_3_‐N (mg L^−1^)	0.32 ± 0.11	0.31 ± 0.12	0.19 ± 0.04	0.33 ± 0.14	4.79	0.006
NH_3_‐N (mg L^−1^)	0.39 ± 0.11	0.19 ± 0.05	0.11 ± 0.04	0.14 ± 0.11	26.48	< 0.001

*Note:* S1, upstream reach; S2, reservoir; S3, dewatered reach; S4, downstream reach. F and *p* values are from one‐way ANOVA testing differences among sites and among seasons, respectively. Shaded *p* values indicate significant differences (*p* < 0.05).

Water temperature ranged from 10.64°C ± 2.69°C in winter to 23.99°C ± 1.47°C in summer (F = 47.21, *p* < 0.001), and dissolved oxygen showed the inverse pattern, from 7.49 ± 0.68 mg L^−1^ in summer to 13.44 ± 1.20 mg L^−1^ in winter (F = 68.06, *p* < 0.001), consistent with temperature‐dependent oxygen solubility. Nutrient concentrations were likewise seasonally structured, with TN, TP, NO_3_‐N, and NH_3_‐N all differing significantly among seasons (all *p* ≤ 0.006). These patterns indicate that the influence of the SHP on the measured environmental variables was expressed primarily through hydrological modification rather than changes in water chemistry, which was dominated by seasonal forcing.

**FIGURE 2:**
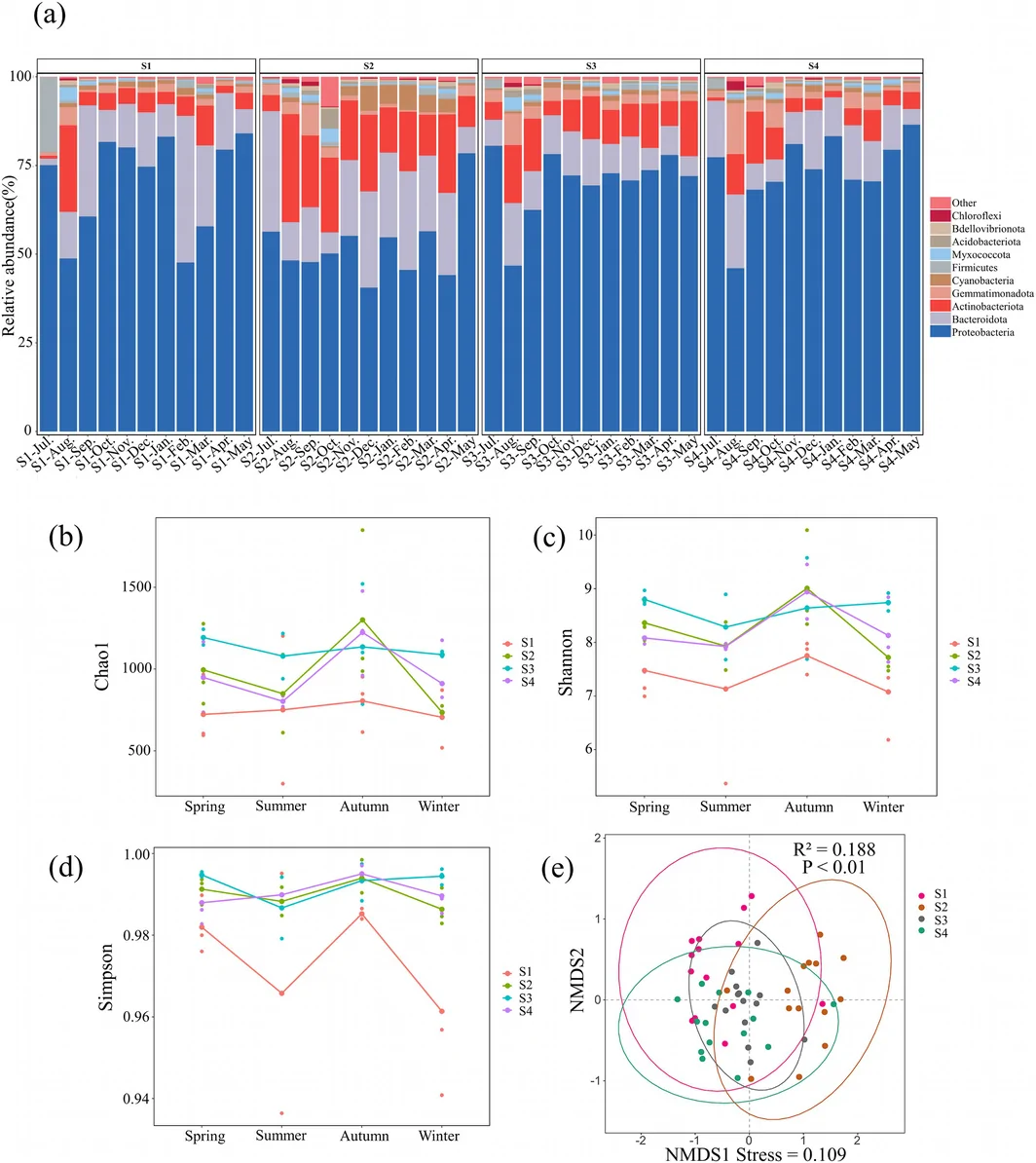
Spatial and seasonal variations in the composition and diversity of benthic biofilm bacterial communities across four sampling sites (S1: Upstream, S2: Reservoir area, S3: Dewatered reach, S4: Downstream). (a) Relative abundance of bacterial communities at the phylum level based on 16S rRNA gene sequencing data. Seasonal and spatial comparisons of alpha diversity indices including (b) Chao1 index (community richness), (c) Shannon index, and (d) Simpson index (community evenness). (e) NMDS ordination plot showing beta diversity patterns of benthic biofilm microbial communities across all sampling sites and seasons, reflecting overall community compositional differences.

A one‐year monitoring survey of the SHP identified a total of 38 phyla, 828 genera, and 1601 species, with the five most abundant phyla being Proteobacteria, Bacteroidota, Actinobacteriota, Gemmatimonadota, and Firmicutes. The Proteobacteria were overwhelmingly dominant, comprising 70%–73% of the community at S1, S3, and S4, but were relatively less abundant at S2 (52%). Bacteroidota and Actinobacteriota reached their highest relative abundances at S2 (22% and 19%, respectively). Alpha diversity differed significantly among sites (Shannon: F = 5.87, *p* = 0.002; Chao1: F = 3.96, *p* = 0.015; Simpson: F = 6.73, *p* = 0.001), with S3 showing the highest values (Shannon 8.65 ± 0.55), followed by S4 (8.30 ± 0.64), S2 (8.29 ± 0.74), and S1 (7.38 ± 0.97). Diversity tended to be lower in summer and winter than in spring and autumn, although this seasonal difference was not statistically significant (Shannon: F = 1.82, *p* = 0.159; Figure 2b–d). The NMDS yielded a stress value of 0.109 (stress < 0.2 indicates good dimensionality reduction), demonstrating reliable representation of community dissimilarities. The PERMANOVA confirmed that site location significantly influenced community composition (*p* < 0.01, R^2^ = 0.188) (Figure 2e). Notably, S2 was distinctly separated from other sites in the NMDS ordination. The NMDS analysis of seasonal variations yielded a stress value of 0.147 (Figure S1), revealing a distinct separation of the summer community from those of other seasons, which was statistically supported (*p* = 0.008, R^2^ = 0.107). A permutation‐based test of multivariate homogeneity (betadisper) further confirmed that the seasonal PERMANOVA result was not biased by uneven sampling months across seasons (F_3,40_ = 0.9507, *p* = 0.416; all pairwise *p* > 0.05).

### 3.2 Niche Characteristics of Benthic Biofilm Microbial Communities Across Seasons

**FIGURE 3:**
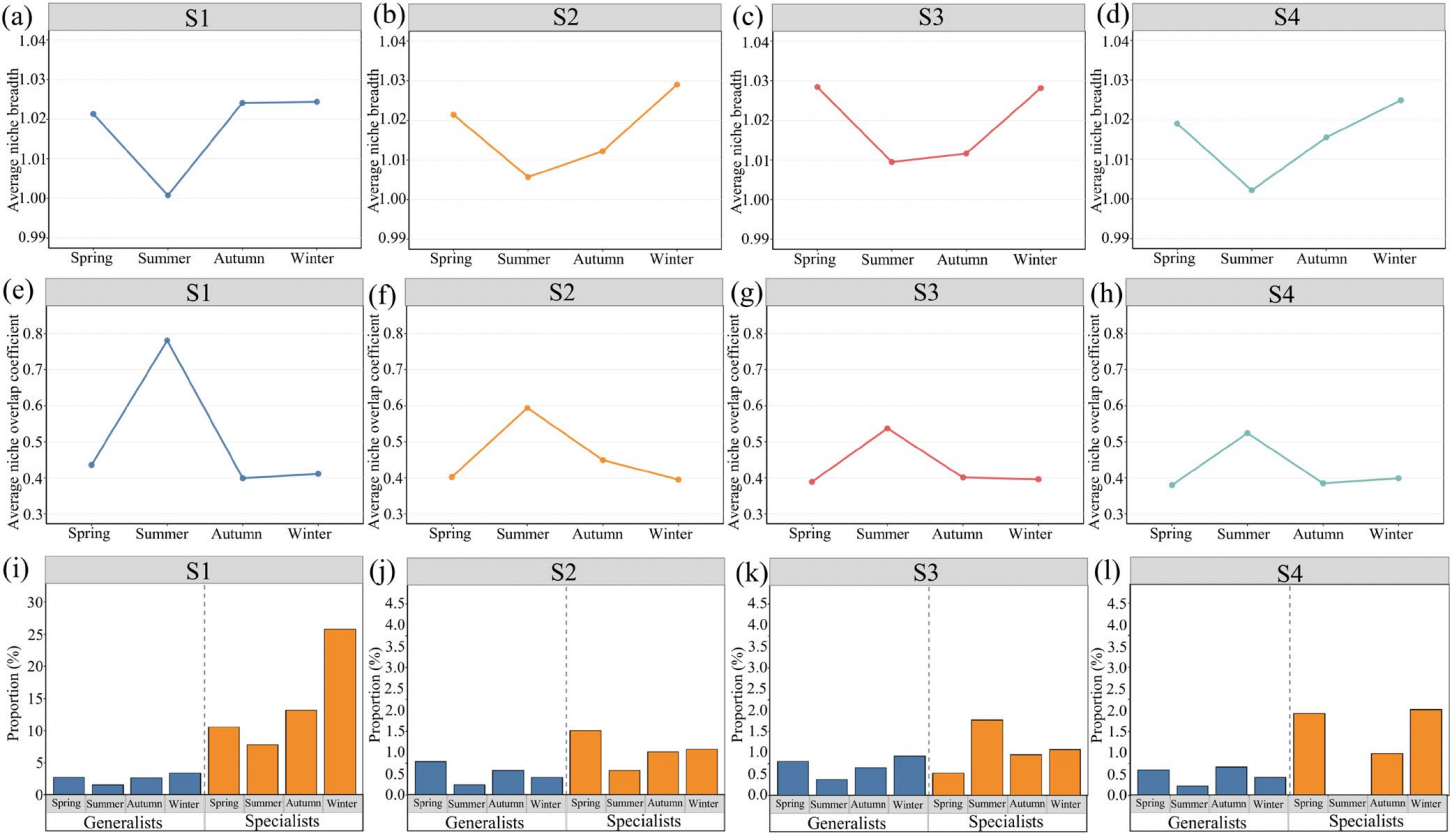
Spatial and seasonal characteristics of niche breadth, niche overlap, and species trophic strategy of benthic biofilm microbial communities across four sampling sites (S1–S4). Average niche breadth for (a) S1, (b) S2, (c) S3, and (d) S4 across different seasons. Average niche overlap coefficient for (e) S1, (f) S2, (g) S3, and (h) S4 across different seasons. Proportional abundance of generalists and specialists for (i) S1, (j) S2, (k) S3, and (l) S4 across different seasons.

The benthic biofilm microbial communities exhibited significant differences in niche breadth, overlap, and differentiation across sampling sites and seasons. The average niche breadth was highest at S3 (1.019) compared to site S1 (1.018), S2 (1.017), and S4 (1.015) (Figure 3c). In contrast, S1 showed the highest average niche overlap coefficient (0.506), indicating stronger interspecific competition among microbial taxa at this site (Figure 3e). On a temporal scale, the mean niche breadth was the narrowest in summer (1.005) and the widest in winter (1.027), whereas the mean niche overlap coefficient was the lowest in winter (0.400) and the highest in summer (0.609). A clear negative correlation (ρ = −0.692) was observed between the niche overlap coefficient and niche breadth across most sites and seasons (Figure S2). However, this pattern was absent at S4 during winter, suggesting that the relationship between species competition and nutrient resources in the S4 community during this season differs from that at other sites. Analysis of the relative abundances of generalists and specialists revealed that specialists were consistently more abundant than generalists across all communities (Figure 3i–l). Specifically, the average abundance of specialists was highest in S1 (14.33%), while the average abundance of generalists was highest in S3 (4.62%), consistent with Hypothesis 1 and 2.

### 3.3 Assembly Processes of Benthic Biofilm Microbial Communities Across Sites and Seasons

**FIGURE 4:**
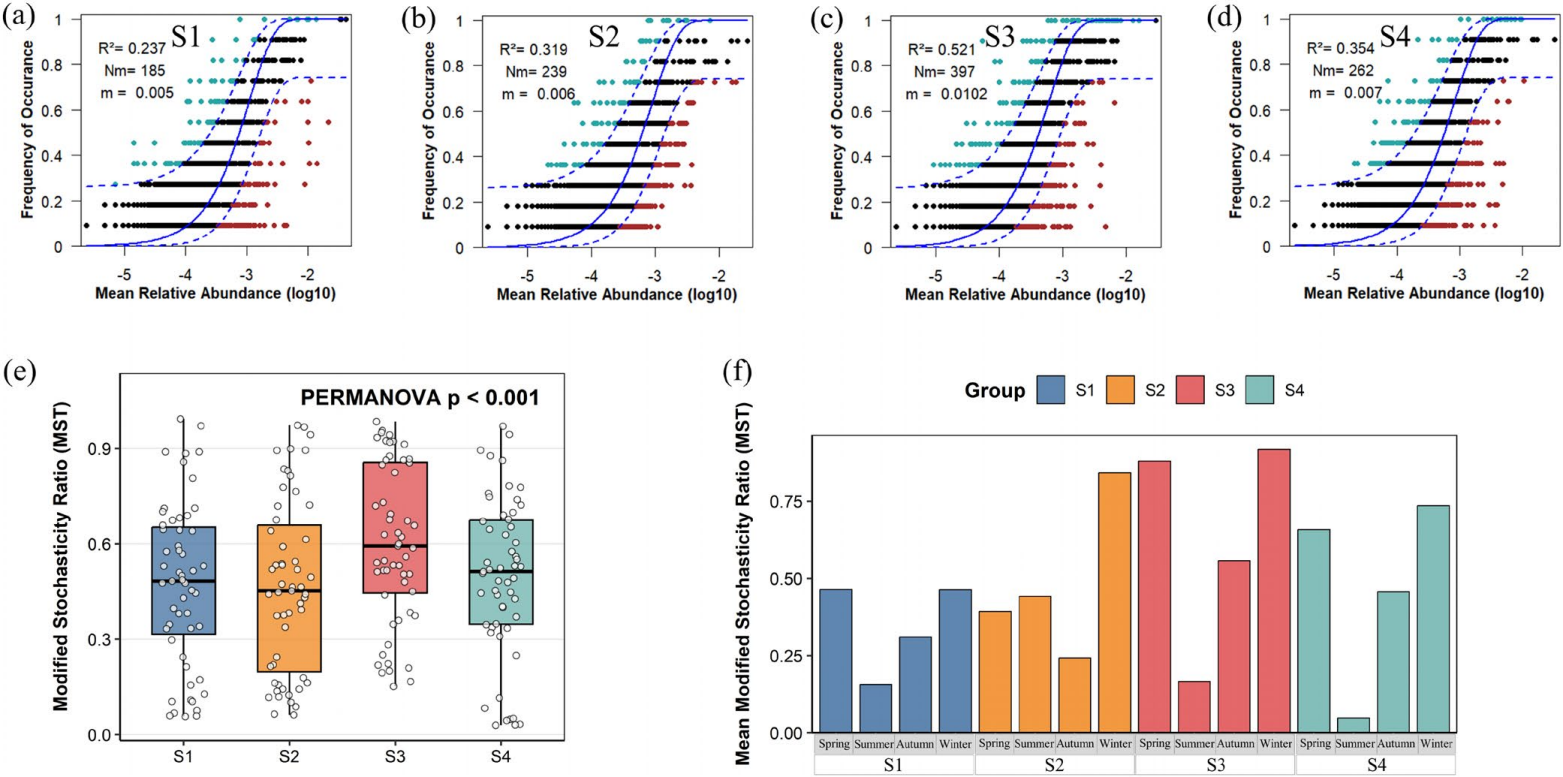
Community assembly processes of benthic biofilm bacteria. Fit of the neutral community model for (a) S1, (b) S2, (c) S3, and (d) S4. Each point represents a bacterial ASV. Points are colored based on whether their occurrence frequency was higher or lower than predicted by the neutral model. The solid black line indicates the predicted occurrence frequency, and the dashed lines represent the 95% confidence interval around the neutral model prediction. (e) MST values across all sites; an MST > 0.5 indicates that stochastic processes dominate community assembly. (f) Seasonal variations in the mean MST for benthic biofilm bacterial communities at different sites.

The neutral community model revealed predictable distribution patterns for bacterial communities (Figure 4), with highest R^2^ value at S3 (0.521) and lowest at S1 (0.237); the values at S2 and S4 were 0.319 and 0.354, respectively. Community dispersal, quantified by the neutral model parameter Nm, was highest in S3 (Nm = 397), followed by S4 (Nm = 262), while S1 exhibited the lowest dispersal level (Nm = 185).

The S3 bacterial community showed a mean MST significantly greater than 50% (PERMANOVA, *p* < 0.001), indicating a dominant role of stochastic assembly processes. In contrast, deterministic processes primarily governed the assembly of microbial communities at other sites. From a temporal perspective, both S2 and S4 exhibited stronger influences of stochastic processes during the dry season (winter), while deterministic processes became more dominant during the wet season (summer).

### 3.4 Site‐Specific Variation in Benthic Biofilm Genes

**FIGURE 5:**
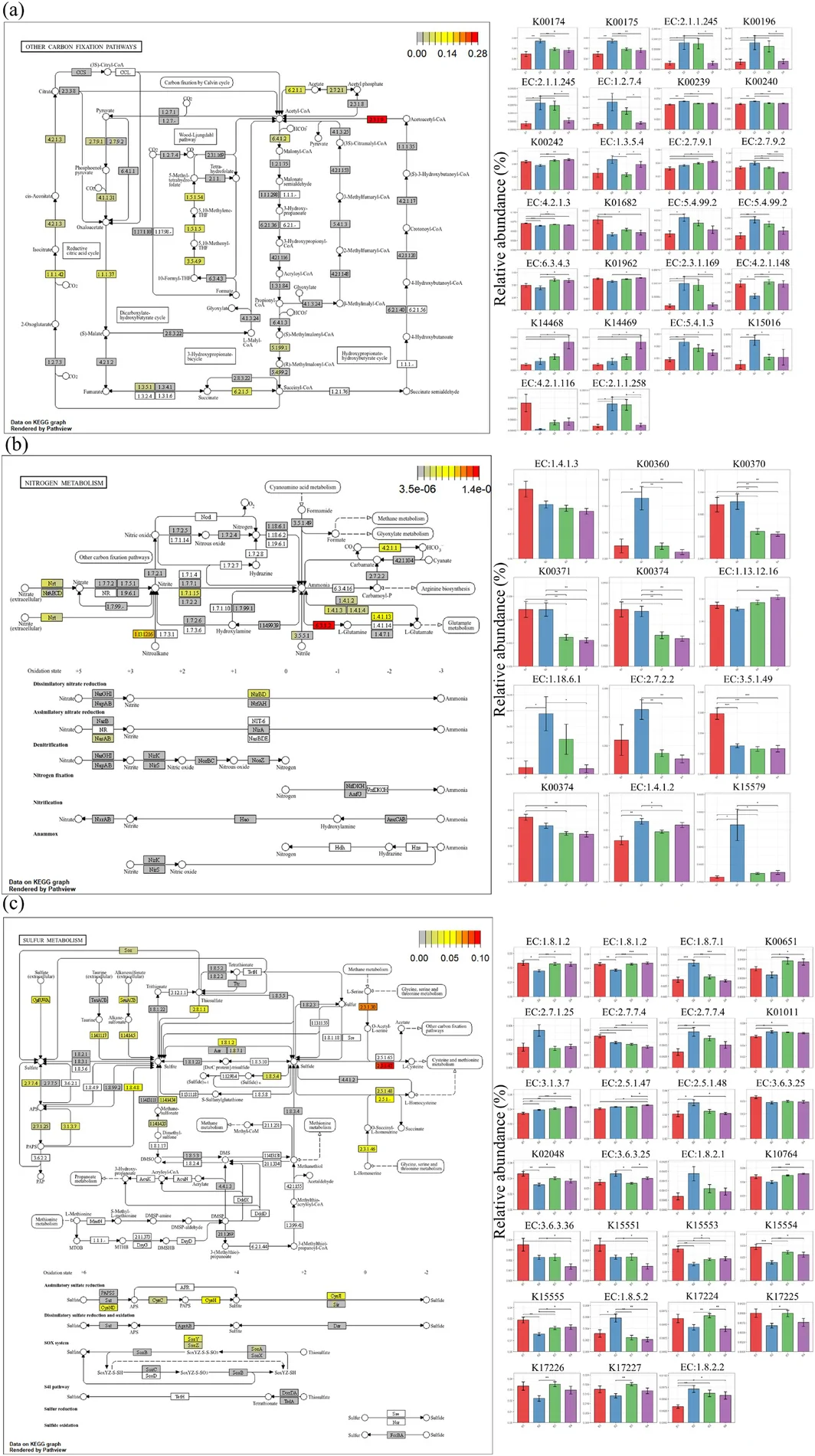
Predicted differential functional gene profiles of carbon, nitrogen, and sulfur metabolic pathways in benthic biofilm microbial communities based on Phylogenetic Investigation of Communities by PICRUSt2 functional prediction. (a) Prokaryotic carbon fixation pathways, (b) nitrogen metabolism pathways, and (c) sulfur metabolism pathways. For each subgraph, the left panel lists core KEGG Level 3 metabolic pathways and corresponding differential functional genes/enzymes involved in biogeochemical cycling. The right bar graphs quantify the enrichment magnitude and significance of pathway‐related differential genes across sampling sites. Different colors in the bar plots represent distinct comparison groups, including upregulated and downregulated functional genes under spatial variation. All functional profiles are computationally predicted from 16S rRNA gene data and do not represent direct metagenomic measurements.

Overall, variations in genes predicted to be associated with carbon, nitrogen, and sulfur cycles were more pronounced between sampling sites than between seasons. Specifically, the gene encoding acetyl‐CoA C‐acetyltransferase (EC:2.3.1.9; K00626), involved in carbon fixation pathways, exhibited the highest predicted abundance across all sampling sites and in all seasons (Figures 5a and S3). Site S2 showed significant enrichment in multiple carbon fixation genes, including pyruvate, water dikinase (EC:2.7.9.2; K01007), acetyl‐CoA synthetase (EC:6.2.1.1; K01907), and methylmalonyl‐CoA mutase (EC:5.4.99.2; K01847). In contrast, S4 exhibited a higher abundance of the acetyl‐CoA carboxylase biotin carboxyl carrier protein gene (EC:6.4.1.2; K02160). Summer showed a lower predicted abundance of the acetyl‐CoA carboxylase biotin carboxyl carrier protein gene (EC:6.4.1.2; K02160).

In nitrogen metabolism pathways (Figures 5b and S4), the most abundant predicted genes across all sites and all seasons were those encoding glutamine synthetase (EC:6.3.1.2; K01915) and nitronate monooxygenase (EC:1.13.12.16; K00459). Site S1 demonstrated significantly higher predicted abundance of glutamate dehydrogenase (EC:1.4.1.3; K00261) and nitrilase (EC:3.5.5.1; K01501). In contrast, the predicted abundance of nitronate monooxygenase (EC:1.13.12.16; K00459) was markedly lower at site S2 and during the summer compared to other sites and seasons.

Regarding sulfur metabolism (Figures 5c and S5), all sites and all seasons were primarily involved in processes such as assimilatory sulfate reduction and sulfur oxidation (SOX system). Genes encoding cysteine synthase (EC:2.5.1.47; K01738) and serine O‐acetyltransferase (EC:2.3.1.30; K00640) showed higher predicted abundances. At S2, genes related to the sulfate/thiosulfate transport system substrate‐binding protein (CysPUWA), sulfite reductase (NADPH) flavoprotein alpha‐component (EC:1.8.1.2; K00380), and sulfonate transport system substrate‐binding protein (SsuACB) were less abundant. Conversely, the gene encoding sulfite reductase (ferredoxin) (EC:1.8.7.1; K00392) was notably higher predicted abundance. In terms of seasonal variation, the predicted abundance of the gene encoding sulfite reductase (ferredoxin) (EC:1.8.7.1; K00392) was significantly lower in spring, while summer showed lower abundance in quinone oxidoreductase (EC:1.8.5.4; K17218).

## 4 Discussion

### 4.1 Seasonal Variation of Biofilm Microbes Under SHP

**FIGURE 6:**
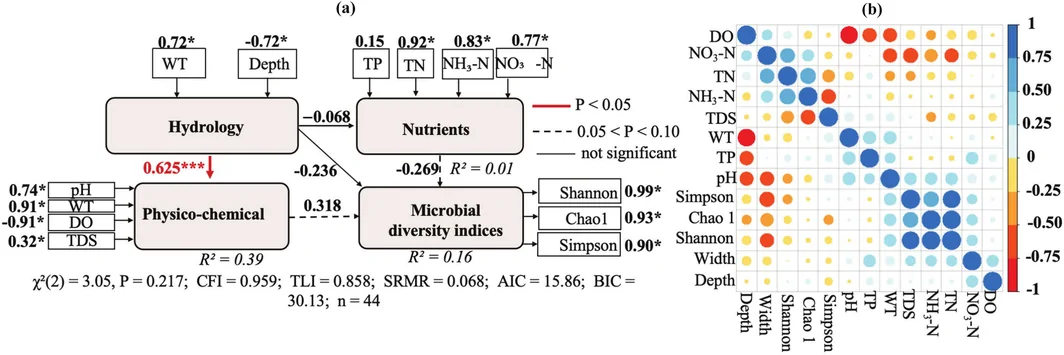
Relationships between hydrology, physico‐chemical conditions, nutrients and microbial diversity in the study river (*n* = 44 site‐month observations). (a) Structural equation model testing the hypothesized causal pathways. Rectangles with rounded corners denote composite variables, each derived as the first principal component of the observed indicators shown in adjacent boxes; values beside indicators are indicator–composite correlations. Numbers on arrows are standardized path coefficients. Red solid arrows indicate significant paths (*p* < 0.05), dashed arrows marginal trends (0.05 < *p* < 0.10), and thin solid arrows non‐significant paths. R^2^ values give the proportion of variance explained for each endogenous variable. Model fit: χ^2^ (2) = 3.05, *p* = 0.217; CFI = 0.959; TLI = 0.858; SRMR = 0.068; AIC = 15.86; BIC = 30.13. (b) Pearson correlation matrix among all measured environmental and diversity variables. The r values are shown in Table S2.

Our findings support the prevalence of Proteobacteria and Bacteroidota in freshwater ecosystems. Existing studies have reported that Proteobacteria and Actinobacteriota can constitute over 50% of the total bacterial community in surface waters (Yu et al. 2014). The dominance of Proteobacteria suggests their crucial role in the functions and processes of riverine ecosystems (Zhang et al. 2015). Furthermore, river depth and width act as key environmental factors influencing bacterial community structure (Röske et al. 2012). This can also be seen in our SEM (Figure 6, Table S3), where river depth and width positively affect microbial abundance. In the present study, by contrast, neither depth nor width was significantly correlated with any microbial diversity index (all |r| ≤ 0.34; Table S2), and the corresponding structural path was not significant (Figure 6a, Table S3). However, previous research indicates that the relative abundance of Proteobacteria is negatively correlated with water depth, whereas that of Bacteroidota shows a positive correlation (Chen et al. 2017). The observed higher abundance of Bacteroidota at S2 is consistent with their known adaptability to the depositional, low‐flow conditions characteristic of impounded reaches. We note that DO did not differ significantly among sites (F = 0.28, *p* = 0.839; Table 1), and that variation in DO in this system was predominantly seasonal rather than spatial; we therefore do not attribute this pattern to site‐level oxygen depletion (Qi et al. 2024). The other species can be adversely affected by the low DO, as shown in the correlation analysis in Figure 6b. Additionally, the capacity of Bacteroidota to degrade complex polysaccharides into bioavailable small molecules may confer a competitive advantage in aquatic environments (Liu et al. 2009). Actinobacteria, capable of degrading recalcitrant compounds like polycyclic aromatic hydrocarbons (Zhu et al. 2019), likely contribute to organic matter decomposition in the S2 benthic biofilms. The dominance of Bacteroidota and Actinobacteriota in reservoir environments, as documented in multiple studies, further corroborates our findings (Qu et al. 2008; Röske et al. 2012).

S1, located in a natural flow regime with relatively low anthropogenic disturbance, exhibited lower bacterial diversity, which may be associated with stronger top‐down biological control (Wang et al. 2023). Previous studies have reported relatively high diversity and taxonomic richness of benthic macroinvertebrates in this region (Lin et al. 2024), suggesting stable environmental conditions that support organisms at higher trophic levels. In contrast, the reservoir area (S2), characterized by low flow velocity, reduced DO, and elevated nutrient levels, appears to support a wider range of bacterial taxa (Wang et al. 2023). S3, situated in the dewatered reach, is influenced by nutrient inputs from ecological water releases, considerable habitat heterogeneity, as well as concurrent anthropogenic activities and allochthonous nutrient inputs from downstream and riparian areas. These combined factors may explain the observed spatial pattern of lower diversity at S1 and higher diversity at S3. The negative correlation between the nutrients and Chao1, Simpson, and Shannon may be because of dewatering (Figure 6b).

Seasonally, concentrated summer precipitation and increased discharge can lead to hydrological homogenization, reducing habitat diversity. Furthermore, the recurrent flood disturbances during the wet season also negatively impact biodiversity (Leung et al. 2012). In winter, low temperatures and low water levels suppress microbial growth (Zhang and Gross 2021), and the seasonal drought in the stream can be characterized as a disturbance regime where water inflow, river discharge, and water availability drop to minimal levels for an extended period (Lake 2003). In contrast, spring and autumn provide more suitable temperatures and hydrological conditions. Being in a comparatively stable state, these transitional seasons are more conducive to supporting microbial community growth and maintaining diversity.

### 4.2 Seasonal Variation of Niche Under SHP Influence

The environmental niche is regarded as an intrinsic filter for microbial taxa and their assembled communities (D'Amen et al. 2017). Niche‐based theory posits that microbial communities are shaped by deterministic abiotic factors (e.g., environmental parameters) and biotic factors (e.g., species interactions), driven by differential habitat preferences and adaptations among microorganisms (Dumbrell et al. 2010; Ofiteru et al. 2010). The variations in niche breadth and overlap reflect changes in available nutrient resources and competitive pressures within the community (Muller 2019; Pianka 1974). Niche breadth can serve as an indicator of a species' tolerance to changing conditions (Malard et al. 2022). Generalists, with their broader environmental tolerance, are more likely to maintain viable populations even under suboptimal conditions (Slatyer et al. 2013; Thuiller et al. 2005). The relatively greater average niche breadth and higher proportion of generalists at S3 suggest that its microbial community possesses enhanced resilience to environmental fluctuations, likely attributable to frequent hydrological disturbances and pronounced habitat heterogeneity in this dewatered reach. According to diffuse competition hypothesis and the niche overlap theory, under resource‐limited conditions, competition among taxa is expected to be inversely related to niche breadth (Hirzel and Le Lay 2008; Pianka 1974). The study area is situated in a forested upstream reach, where the overall oligotrophic status and limited resource availability provide an ecological context conducive to the manifestation of such competitive mechanisms. Microbial communities often respond to environmental stress and interspecific competition by differentiating into generalists and specialists, thereby enhancing their overall survival capacity (Berg, Kiers, et al. 2010; Van Tienderen 1991). Previous studies have reported that habitats are often dominated by specialist taxa (Logares et al. 2013; Mariadassou et al. 2015), meaning specialists frequently outnumber generalists in ecological communities, a pattern consistent with our findings across all sampled sites.

### 4.3 Stochastic and Deterministic Assembly Processes of Benthic Biofilm Microbial Communities

Niche‐based and neutral processes jointly govern the assembly of microbial communities (Dini‐Andreote et al. 2015; Liao et al. 2016). Deterministic processes primarily involve ecological selection that influences bacterial fitness, thereby shaping community composition and structure (Zhou and Ning 2017). In contrast, stochastic processes include probabilistic dispersal and random changes in species relative abundance (ecological drift), which are not driven by environmentally determined fitness (Chase 2007; Chase and Myers 2011). The dominance of deterministic processes at S1 indicates that its community structure is largely regulated by environmental filtering and interspecific interactions. This aligns with previous findings that macroinvertebrate communities in upstream sections of SHPs are also governed predominantly by deterministic processes (Lin et al. 2024). Collectively, these results suggest that S1, which is situated in a natural flow regime, experiences stable habitat conditions with relatively low anthropogenic disturbance, where environmental factors strongly filter species composition. In contrast to macroinvertebrate communities, which have been reported (Lin et al. 2024) to be strongly influenced by stochastic processes in reservoir areas (S2), the benthic biofilm microbial communities in our study showed higher stochasticity at S3. This may be attributed to ecological water releases and high environmental heterogeneity in the dewatered reach. Such environmental variability can weaken niche‐based selection while enhancing the roles of dispersal and random colonization, thereby promoting the prevalence of generalist species (Székely et al. 2013).

It is noteworthy that generalists are considered to play an important evolutionary role in maintaining taxonomic diversity, offering advantages in speciation rates and community persistence (Pandit et al. 2009). Some studies have proposed that the assembly of specialist‐dominated communities is primarily driven by deterministic processes, whereas neutral processes are more critical for generalist‐dominated communities (Liao et al. 2016; Pandit et al. 2009). This perspective appears consistent with our results: S1, which is more influenced by deterministic processes, hosted a higher abundance of specialists. A higher proportion of generalists appeared in S3, where stochastic processes predominated. At the seasonal scale, S2 and S4 exhibited dynamic shifts in the balance of assembly processes. During the wet season, seasonal precipitation leads to a sharp increase in basin discharge. The limited storage capacity of the small hydropower plant means that high runoff and rapid flow velocities can cause water to overtop the dam and surge into the dewatered reach. This can homogenize water conditions across the SHP‐influenced reach (de Oliveira and Margis 2015), strengthening environmental filtering and elevating the dominance of deterministic processes. In contrast, during the dry season, reduced water levels and slower flow rates intensify dispersal limitation (Li et al. 2019). As a process that can enhance stochasticity and obscure the relationship between environmental variables and microbial community composition, dispersal limitation prevents microorganisms from reaching new habitats (Evans et al. 2017), potentially restricts microbial dispersal, and enhances local drift, thereby increasing the contribution of stochastic processes.

### 4.4 Predicted Functional Potential in Benthic Biofilm Microbial Communities Across Sites Under the SHP

Environmental variation may influence microbial structure and predicted function, potentially triggering adaptive changes in predicted functional potential and metabolite synthesis (Kashtan et al. 2014; Shi and Falkowski 2008). Six autotrophic CO_2_ fixation pathways are currently known, three of which—the reductive tricarboxylic acid (rTCA) cycle, the reductive acetyl‐CoA pathway, and the 3‐hydroxypropionate cycle—have been reported in bacteria (Berg, Kockelkorn, et al. 2010). Both methylmalonyl‐CoA mutase, which showed higher predicted abundance at S2, and the acetyl‐CoA carboxylase biotin carboxyl carrier protein, enriched at S4, are predicted to belong to the 3‐hydroxypropionate cycle. However, genes enriched at S2 also participate in other carbon fixation pathways, suggesting that microbes at S2 may employ multiple carbon fixation strategies to cope with the hydrologically fluctuating reservoir environment. Glutamine synthetase was relatively abundant across all sites. Glutamine synthetase serves as the primary assimilatory enzyme for ammonia derived from nitrogen fixation, nitrate, or ammonium nutrition, and it reassimilates ammonia released from photorespiration, protein degradation, and nitrogen transport compound breakdown (Miflin and Habash 2002).

Consequently, Glutamine synthetase plays a central role in nitrogen metabolism, and its activity varies with environmental conditions. Nitronate monooxygenase, a key enzyme in nitroalkane catabolism involved in defense mechanisms across diverse organisms, may assist benthic biofilm microbes at various sites in detoxifying harmful compounds (Torres‐Guzman et al. 2021). Glutamate and ammonium are critical nitrogen donors for nearly all cellular biosynthetic reactions and represent preferred, readily utilized nitrogen sources for microbial communities (Wang et al. 2020; Wong et al. 2008). The higher abundance of glutamate dehydrogenase at the oligotrophic S1 suggests that the microbial community may rely on efficient internal recycling of organic nitrogen resources. Under sulfur‐sufficient conditions, metabolites such as cysteine and glutathione are predicted to act as regulators of sulfur uptake and assimilation at the predicted functional potential level. Under sulfur limitation, decreased levels of these compounds lift this repression, leading to enhanced transporter activity and maximized sulfate uptake (Hesse et al. 2004; Smith et al. 1997; Takahashi et al. 2000). In sulfur metabolism, the significant downregulation of sulfate transporter genes at S2 may be related to slow water movement in the reservoir area, which promotes nutrient retention and likely results in relatively sufficient sulfur availability.

## 5 Conclusion

This case study demonstrates that a diversion‐type SHP can significantly alter the community structure, assembly processes, and predicted metabolic functions of benthic biofilm microbes in a subtropical montane river. Among the longitudinal sites, the most pronounced ecological shifts were observed between the reservoir and the dewatered reach, where flow reduction was associated with increased microbial diversity, a transition from deterministic to stochastic community assembly, and enhanced functional gene enrichment related to carbon and nitrogen cycling. Seasonal hydrological fluctuations further modulated these patterns, indicating that both spatial and temporal dimensions are essential for assessing SHP impacts. These findings carry two important ecological implications. First, the shift from deterministic to stochastic assembly in the dewatered reach suggests that flow reduction may weaken environmental filtering, potentially altering the resilience and stability of benthic biofilm communities. Second, the concurrent enrichment of biogeochemical cycling genes implies that the reservoir could affect nutrient processing dynamics in these river systems, with possible consequences for downstream ecosystem functioning. As this study was based on a single SHP and observational data, these findings should be interpreted as hypothesis‐generating rather than conclusive. Future studies incorporating a broader range of SHP types, longer‐term monitoring, and eukaryotic microbial analysis will be needed to comprehensively assess the ecological impacts of SHPs on benthic biofilm microbial communities. Such efforts would provide a more robust scientific foundation for ecological impact assessment and sustainable management of small hydropower systems from a perspective of watershed view (Cai 2026; Xu 2026).

## Author Contributions

**Huimin Gao:** conceptualization (equal), data curation (equal), writing – original draft (equal). **Sangar Khan:** methodology (equal), visualization (equal), writing – review and editing (equal). **Xinxin Qi:** methodology (equal), visualization (equal). **Zongwei Lin:** investigation (equal), software (equal). **Guohao Liu:** data curation (equal), investigation (equal). **Yuanyuan Lv:** data curation (equal), investigation (equal). **Min Zhang:** investigation (equal), writing – review and editing (equal). **Zhijun Xia:** writing – review and editing (equal). **Naicheng Wu:** conceptualization (equal), data curation (equal), funding acquisition (equal), methodology (equal), supervision (equal), writing – review and editing (equal).

## Funding

This study was supported by grants from the National Natural Science Foundation of China (Nos. 52279068, 42350410434). MZ was supported by the National Key Research and Development Program of China (2024YFC3213800), State Key Laboratory of Water Cycle and Water Security (SKL2025RCPY07).

## Conflicts of Interest

The authors declare no conflicts of interest.

## Supporting information


**Figure S1:** Non‐metric multidimensional scaling (NMDS)plot illustrating beta diversity patterns among benthic biofilm microbial communities from different seasons.
**Figure S2:** Results of Spearman correlation analysis of benthic biofilm community niche breadth and niche overlap coefficient across seasons and sampling sites.
**Figure S3:** Differential abundance analysis of genes/enzymes involved in prokaryotic carbon fixation pathways in KEGG for benthic biofilm microbial communities across seasons.
**Figure S4:** Differential abundance analysis of genes/enzymes involved in nitrogen metabolism pathways in KEGG for benthic biofilm microbial communities across seasons.
**Figure S5:** Differential abundance analysis of genes/enzymes involved in sulfur metabolism pathways in KEGG for benthic biofilm microbial communities across seasons.
**Table S1:** Variations in environmental indices—including hydrological, physicochemical, and nutrient parameters—across the four sampling sites over different months.
**Table S2:** Coefficient of determination (R^2^) matrix for hydrological, physico‐chemical, nutrient and microbial diversity variables.
**Table S3:** Standardized path coefficients, indicator loadings and assumption diagnostics for the structural equation model (*n* = 44).
**Table S4:** Sensitivity analysis comparing the structural equation model fitted to the full dataset and to complete cases only.

## Data Availability

Raw sequence data were submitted to the NCBI Sequence Read Archive under BioProject PRJNA 1454217. All the R codes for the analysis are available from Github.
