# Supplementary material for: Seasonal Effect of a Small Hydropower Plant on Benthic Biofilm Microbial Community Structure and Predicted Functional Potential
Source: Ecol Evol. 2026 Sep 8;16(9):e74330. doi: 10.1002/ece3.74330 (PMC13553146; doi:10.1002/ece3.74330)
Supplement: Supplementary file 1 [file ECE3-16-e74330-s001.docx]

**Supplementary materials**

Seasonal Effect of a Small Hydropower Plant on Benthic Biofilm Microbial Community Structure and Predicted Functional Potential

Huimin Gao^a#^, Sangar Khan^a#^, Xinxin Qi^a^, Zongwei Lin^b^, Guohao Liu^b^, Yuanyuan Lv^c^, Min Zhang^d^, Zhijun Xia^e^, Naicheng Wu^af^*

^#^ This author contributed equally as the first author.

^*^ Corresponding author:

Naicheng Wu ([naichengwu88@gmail.com](mailto:naichengwu88@gmail.com); wunaicheng@nbu.edu.cn)

**Supplementary Figures**

Fig. S1. Non-metric multidimensional scaling (NMDS)plot illustrating beta diversity patterns among benthic biofilm microbial communities from different seasons.

Fig. S2. Results of Spearman correlation analysis of benthic biofilm community niche breadth and niche overlap coefficient across seasons and sampling sites.

Fig. S3. Differential abundance analysis of genes/enzymes involved in prokaryotic carbon fixation pathways in KEGG for benthic biofilm microbial communities across seasons.

Fig. S4. Differential abundance analysis of genes/enzymes involved in nitrogen metabolism pathways in KEGG for benthic biofilm microbial communities across seasons.

Fig. S5. Differential abundance analysis of genes/enzymes involved in sulfur metabolism pathways in KEGG for benthic biofilm microbial communities across seasons.

**Tables**

Table S1. Variations in environmental indices—including hydrological, physicochemical, and nutrient parameters—across the four sampling sites over different months.

| 4 | Season | Velocity (m/s) | Width (m) | Depth (cm) | pH | WT (◦C) | DO (mg/L) | TDS (mg/L) | TN (mg/L) | TP (mg/L) | NO_3_-N (mg/L) | NH_3_-N (mg/L) |
| --- | --- | --- | --- | --- | --- | --- | --- | --- | --- | --- | --- | --- |
| S1-Jul. | Summer | NA | 7.3 | 45 | 7.39 | 25.0 | 8.40 | 14.30 | 0.65 | 0.09 | 0.47 | 0.17 |
| S2-Jul. | Summer | NA | 15.1 | 53 | 6.85 | 25.3 | 7.23 | 14.30 | 0.48 | 0.08 | 0.26 | 0.15 |
| S3-Jul. | Summer | NA | 19.5 | 43 | 6.94 | 24.1 | 7.59 | 14.95 | 1.18 | 0.12 | 0.33 | 0.26 |
| S4-Jul. | Summer | NA | 4.8 | 15 | 7.03 | 26.4 | 8.50 | 15.60 | 0.82 | 0.06 | 0.49 | 0.28 |
| S1-Aug. | Summer | 0.689 | 7.3 | 53 | 7.09 | 22.7 | 6.66 | 12.35 | 0.41 | 0.07 | 0.18 | 0.20 |
| S2-Aug. | Summer | 0 | 15.1 | NA | 7.37 | 22.6 | 7.22 | 11.70 | 0.39 | 0.07 | 0.21 | 0.17 |
| S3-Aug. | Summer | 0 | 19.5 | NA | 7.29 | 22.4 | 6.75 | 11.70 | 0.47 | 0.08 | 0.26 | 0.15 |
| S4-Aug. | Summer | 0.421 | 4.8 | 32 | 7.40 | 23.4 | 7.61 | 13.00 | 0.45 | 0.09 | 0.26 | 0.17 |
| S1-Sep. | Autumn | NA | 7.3 | 35 | 6.77 | 21.4 | 10.09 | 11.05 | 0.45 | 0.04 | 0.23 | 0.15 |
| S2-Sep. | Autumn | NA | 15.1 | 75 | 7.50 | 19.6 | 9.28 | 11.70 | 0.63 | 0.09 | 0.23 | 0.18 |
| S3-Sep. | Autumn | NA | 19.5 | 35 | 7.61 | 21.0 | 7.61 | 11.70 | 0.36 | 0.03 | 0.17 | 0.17 |
| S4-Sep. | Autumn | NA | 4.8 | 32 | 7.63 | 22.0 | 9.81 | 12.35 | 0.35 | 0.04 | 0.18 | 0.14 |
| S1-Oct. | Autumn | 0.480 | 3.5 | 40 | 7.19 | 18.9 | 10.76 | 13.65 | 0.40 | 0.06 | 0.28 | 0.08 |
| S2-Oct. | Autumn | 0 | 13.8 | NA | 7.69 | 18.9 | 8.04 | 13.65 | 0.39 | 0.09 | 0.17 | 0.10 |
| S3-Oct. | Autumn | 0 | 17.8 | NA | 7.62 | 18.8 | 8.84 | 13.65 | 0.86 | 0.08 | 0.21 | 0.09 |
| S4-Oct. | Autumn | 0.013 | 2.1 | 21 | 7.56 | 18.9 | 9.62 | 14.30 | 0.28 | 0.06 | 0.18 | 0.09 |
| S1-Nov. | Autumn | 0.706 | 3.3 | 15 | 6.84 | 17.0 | 8.76 | 16.25 | 0.32 | 0.07 | 0.18 | 0.09 |
| S2-Nov. | Autumn | 0 | 13.6 | 75 | 7.78 | 17.0 | 8.44 | 14.30 | 0.28 | 0.09 | 0.15 | 0.08 |
| S3-Nov. | Autumn | 0 | 16.5 | 12 | 7.74 | 17.1 | 8.49 | 14.95 | 0.40 | 0.10 | 0.16 | 0.09 |
| S4-Nov. | Autumn | 0.088 | 2.1 | 22 | 7.16 | 17.5 | 9.26 | 14.95 | 0.39 | 0.04 | 0.11 | 0.08 |
| S1-Dec. | Winter | 0.864 | 2.3 | 23 | 6.57 | 14.6 | 12.03 | 14.30 | 0.64 | 0.05 | 0.34 | 0.07 |
| S2-Dec. | Winter | 0 | 15.1 | 83 | 7.28 | 14.2 | 14.41 | 14.95 | 0.32 | 0.05 | 0.20 | 0.06 |
| S3-Dec. | Winter | 0 | 13.2 | 49 | 7.37 | 13.7 | 12.68 | 14.95 | 0.78 | 0.04 | 0.30 | 0.09 |
| S4-Dec. | Winter | 0.192 | 2.2 | 27 | 7.65 | 14.2 | 12.03 | 14.30 | 0.54 | 0.03 | 0.19 | 0.08 |
| S1-Jan. | Winter | 0.391 | 3.9 | 26 | 6.15 | 10.4 | 13.96 | 11.70 | 0.62 | 0.04 | 0.55 | 0.05 |
| S2-Jan. | Winter | 0 | 9.7 | 54 | 6.67 | 9.7 | 13.3 | 14.95 | 0.70 | 0.05 | 0.38 | 0.07 |
| S3-Jan. | Winter | 0 | 18.7 | 43 | 6.8 | 9 | 13.03 | 14.95 | 0.50 | 0.05 | 0.33 | 0.07 |
| S4-Jan. | Winter | 0.047 | 2.3 | 24 | 6.47 | 8.8 | 16.25 | 14.95 | 0.48 | 0.03 | 0.29 | 0.06 |
| S1-Feb. | Winter | 0.586 | 4.1 | 46 | 6.10 | 8.3 | 14.43 | 10.40 | 1.68 | 0.05 | 0.62 | 0.37 |
| S2-Feb. | Winter | 0.283 | 12.6 | 73 | 6.77 | 8.3 | 13.66 | 10.40 | 1.03 | 0.07 | 0.23 | 0.25 |
| S3-Feb. | Winter | 0 | 17.8 | 23 | 7.12 | 8.4 | 12.76 | 10.40 | 0.89 | 0.07 | 0.21 | 0.20 |
| S4-Feb. | Winter | 0.576 | 3.2 | 21 | 7.35 | 8.1 | 12.69 | 10.40 | 1.11 | 0.05 | 0.29 | 0.27 |
| S1-Mar. | Spring | 0.158 | 5.6 | 38 | 6.14 | 13.7 | 10.94 | 10.40 | 0.97 | 0.06 | 0.44 | 0.49 |
| S2-Mar. | Spring | 0 | 14.0 | 55 | 6.96 | 13.6 | 8.62 | 11.05 | 0.85 | 0.05 | 0.23 | 0.43 |
| S3-Mar. | Spring | 0 | 17.8 | 32 | 6.86 | 13.2 | 10.57 | 10.40 | 0.76 | 0.06 | 0.20 | 0.42 |
| S4-Mar. | Spring | 0.017 | 1.4 | 58 | 7.17 | 13.7 | 8.84 | 11.70 | 0.80 | 0.03 | 0.22 | 0.46 |
| S1-Apr. | Spring | 0.597 | 6.1 | 20 | 6.47 | 17.20 | 11.09 | 11.05 | 0.95 | 0.06 | 0.48 | 0.47 |
| S2-Apr. | Spring | 0 | 13.8 | 65 | 7.46 | 17.2 | 9.60 | 11.05 | 1.06 | 0.07 | 0.40 | 0.39 |
| S3-Apr. | Spring | 0 | 17.7 | 42 | 7.27 | 17.0 | 9.98 | 11.70 | 0.88 | 0.05 | 0.33 | 0.33 |
| S4-Apr. | Spring | 0.087 | 2.3 | 42 | 7.54 | 18.6 | 9.73 | 11.70 | 1.02 | 0.05 | 0.23 | 0.30 |
| S1-May | Spring | 0.635 | 5.1 | 45 | 7.35 | 22.1 | 8.18 | 13.00 | 1.21 | 0.08 | 0.42 | 0.60 |
| S2-May | Spring | 0 | 13.2 | 29 | 7.78 | 21.7 | 8.24 | 13.00 | 1.11 | 0.10 | 0.44 | 0.33 |
| S3-May | Spring | 0 | 17.7 | 38 | 7.38 | 21.2 | 8.27 | 13.00 | 1.13 | 0.08 | 0.22 | 0.23 |
| S4-May | Spring | 0.167 | 2.1 | 21 | 7.41 | 21.6 | 9.61 | 13.65 | 0.94 | 0.06 | 0.25 | 0.26 |

**Table S2.** Coefficient of determination (R²) matrix for hydrological, physico-chemical, nutrient and microbial diversity variables.

|  | Width | Depth | pH | WT | DO | TDS | TN | TP | NO₃-N | NH₃-N | Chao1 | Shannon | Simpson |
| --- | --- | --- | --- | --- | --- | --- | --- | --- | --- | --- | --- | --- | --- |
| Width | 1.00 | 0.16 | 0.35 | 0.00 | 0.48 | 0.10 | 0.21 | 0.18 | 0.00 | 0.12 | 0.08 | 0.07 | 0.09 |
| Depth | 0.16 | 1.00 | 0.10 | 0.01 | 0.00 | 0.19 | 0.08 | 0.11 | 0.07 | 0.10 | 0.00 | 0.00 | 0.00 |
| pH | 0.35 | 0.10 | 1.00 | 0.00 | 0.24 | 0.00 | 0.01 | 0.19 | 0.09 | 0.00 | 0.31 | 0.36 | 0.53 |
| WT | 0.00 | 0.01 | 0.00 | 1.00 | 0.07 | 0.77 | 0.01 | 0.37 | 0.14 | 0.58 | 0.20 | 0.16 | 0.07 |
| DO | 0.48 | 0.00 | 0.24 | 0.07 | 1.00 | 0.03 | 0.00 | 0.09 | 0.36 | 0.00 | 0.15 | 0.17 | 0.20 |
| TDS | 0.10 | 0.19 | 0.00 | 0.77 | 0.03 | 1.00 | 0.08 | 0.14 | 0.19 | 0.72 | 0.14 | 0.11 | 0.04 |
| TN | 0.21 | 0.08 | 0.01 | 0.01 | 0.00 | 0.08 | 1.00 | 0.06 | 0.14 | 0.03 | 0.07 | 0.10 | 0.04 |
| TP | 0.18 | 0.11 | 0.19 | 0.37 | 0.09 | 0.14 | 0.06 | 1.00 | 0.00 | 0.10 | 0.01 | 0.01 | 0.00 |
| NO₃-N | 0.00 | 0.07 | 0.09 | 0.14 | 0.36 | 0.19 | 0.14 | 0.00 | 1.00 | 0.07 | 0.07 | 0.12 | 0.22 |
| NH₃-N | 0.12 | 0.10 | 0.00 | 0.58 | 0.00 | 0.72 | 0.03 | 0.10 | 0.07 | 1.00 | 0.11 | 0.11 | 0.07 |
| Chao1 | 0.08 | 0.00 | 0.31 | 0.20 | 0.15 | 0.14 | 0.07 | 0.01 | 0.07 | 0.11 | 1.00 | 0.97 | 0.78 |
| Shannon | 0.07 | 0.00 | 0.36 | 0.16 | 0.17 | 0.11 | 0.10 | 0.01 | 0.12 | 0.11 | 0.97 | 1.00 | 0.89 |
| Simpson | 0.09 | 0.00 | 0.53 | 0.07 | 0.20 | 0.04 | 0.04 | 0.00 | 0.22 | 0.07 | 0.78 | 0.89 | 1.00 |

**Table S3.** Standardized path coefficients, indicator loadings and assumption diagnostics for the structural equation model (n = 44).

**(a) Structural paths**

| Response | Predictor | Std. β | SE | t | P |
| --- | --- | --- | --- | --- | --- |
| Physico-chemical | Hydrology | 0.625 | 0.120 | 5.19 | < 0.001*** |
| Nutrients | Hydrology | −0.068 | 0.154 | −0.44 | 0.661 |
| Microbial diversity | Hydrology | −0.232 | 0.187 | −1.24 | 0.221 |
| Microbial diversity | Nutrients | −0.265 | 0.150 | −1.76 | 0.085 |
| Microbial diversity | Physico-chemical | 0.313 | 0.192 | 1.63 | 0.111 |
| *R² (explained variance)* | Physico-chemical | 0.391 | | | |
|  | Nutrients | 0.005 | | | |
|  | Microbial diversity | 0.163 | | | |

**(b) Indicator loadings**

| Composite | Indicator | Loading (r) | P |
| --- | --- | --- | --- |
| Hydrology | River width | 0.717 | < 0.001*** |
|  | Depth | −0.717 | < 0.001*** |
| Nutrients | TP | 0.146 | 0.344 |
|  | TN | 0.919 | < 0.001*** |
|  | NH_3_-N | 0.829 | < 0.001*** |
|  | NO₃-N | 0.765 | < 0.001*** |
| Physico-chemical | pH | 0.737 | < 0.001*** |
|  | Water temperature | 0.908 | < 0.001*** |
|  | Dissolved oxygen | −0.905 | < 0.001*** |
|  | TDS | 0.319 | 0.035* |
| Microbial diversity | Shannon | 0.995 | < 0.001*** |
|  | Chao1 | 0.927 | < 0.001*** |
|  | Simpson | 0.895 | < 0.001*** |

**(c) Assumption diagnostics**

| Assumption | Test / diagnostic | Result |
| --- | --- | --- |
| Linearity | Residual vs. fitted plots | No curvature |
| Multicollinearity | Variance inflation factor | 1.08 – 1.76 |
| Residual normality (Physico-chemical) | Shapiro–Wilk | W = 0.962, P = 0.160 |
| Residual normality (Nutrients) | Shapiro–Wilk | W = 0.937, P = 0.019 |
| Residual normality (Diversity) | Shapiro–Wilk | W = 0.928, P = 0.009 |

*Composite variables were derived as the first principal component of their constituent indicators. Loadings are Pearson correlations between each indicator and its composite. Model fit: χ²(2) = 3.05, P = 0.217; CFI = 0.959; TLI = 0.858; SRMR = 0.068; AIC = 15.86; BIC = 30.13. *P < 0.05; **P < 0.01; ***P < 0.001.*

**Table S4.** Sensitivity analysis comparing the structural equation model fitted to the full dataset and to complete cases only.

| Path | Std. β | P | Std. β | P |
| --- | --- | --- | --- | --- |
|  | Full dataset (n = 44) | | Complete cases (n = 40) | |
| Hydrology → Physico-chemical | 0.625 | < 0.001 | 0.645 | < 0.001 |
| Hydrology → Nutrients | −0.068 | 0.651 | −0.042 | 0.789 |
| Hydrology → Microbial diversity | −0.236 | 0.189 | −0.224 | 0.251 |
| Nutrients → Microbial diversity | −0.269 | 0.056 | −0.249 | 0.096 |
| Physico-chemical → Microbial diversity | 0.318 | 0.077 | 0.276 | 0.157 |
| *χ²* | 3.05 | | 1.37 | |
| *P (χ²)* | 0.217 | | 0.503 | |
| *CFI* | 0.959 | | 1.000 | |
| *SRMR* | 0.068 | | 0.047 | |

*River depth was not recorded at S2 and S3 in August and October (four of 44 site-month records). The complete-case analysis excludes these four records. All path coefficients retained their sign and significance classification under both treatments, indicating that the treatment of missing depth values did not materially influence the results.*
